# THE ROLE OF SELF-EFFICACY IN ADVANCE CARE PLANNING: DOES RACE AND ETHNICITY AFFECT THIS RELATIONSHIP?

**DOI:** 10.1093/geroni/igad104.3662

**Published:** 2023-12-21

**Authors:** Jessica Yauk, Debra Dobbs

**Affiliations:** University of South Florida, Tampa, Florida, United States; University of South Florida, Tampa, Florida, United States

## Abstract

Advance care planning (ACP) has been identified as essential in improving end-of-life patient satisfaction and goal-concordant care. Despite the benefits of ACP, rates remain particularly low among marginalized populations. Marginalized populations are also at an increased risk of receiving care incongruent with their wishes and reporting low-quality end-of-life care. ACP is a complex process where individuals must decide to plan and implement those plans. Because the ACP process is complex, an individual’s self-efficacy- or belief that they can perform a particular task, may be an important component when predicting ACP completion. Using data from the 2018 wave of the Health and Retirement Study (HRS), the current study aimed to understand how two facets of self-efficacy- mastery and constraint-, related to varying levels of ACP completion (living will, durable power of attorney for healthcare, and advance care planning discussions (DPAHC)). Furthermore, we looked at the possible moderating effects of race/ethnicity between self-efficacy and ACP. Results indicated that Black and Hispanic older adults are significantly less likely to complete all types of ACP. Furthermore, higher self-reported constraint- was significantly related to lower completion of ACP discussions (OR = 0.9, p= .05). Neither mastery or constraint were related to completion of a living will or DPAHC. Race/ethnicity was not found to moderate the relationship between self-efficacy and any level of ACP. Individual variables may be less important than community level variables or life-long discrimination when assessing completion of ACP among marginalized populations.

